# Lymphatic Imaging and Intervention in Congenital Heart Disease

**DOI:** 10.1016/j.jscai.2023.101174

**Published:** 2023-09-27

**Authors:** Christoph Bauer, Mario Scala, Jonathan J. Rome, Gerald Tulzer, Yoav Dori

**Affiliations:** a Department of Paediatric Cardiology, Kepler University Hospital GmbH, Linz, Austria; b Johannes Kepler University Linz, Linz, Austria; c Central Radiology Institute, Kepler University Hospital GmbH, Linz, Austria; d Department of Cardiology, Jill and Mark Fishman Center for Lymphatic Disorders, Children's Hospital of Philadelphia, Philadelphia, Pennsylvania

**Keywords:** congenital heart disease, dynamic contrast magnetic resonance lymphangiography, lymphatic insufficiency, lymphatic interventional techniques, plastic bronchitis, protein-losing enteropathy

## Abstract

The lymphatic system plays a central role in some of the most devastating complications associated with congenital heart defects. Diseases like protein-losing enteropathy, plastic bronchitis, postoperative chylothorax, and chylous ascites are now proven to be lymphatic in origin. Novel imaging modalities, most notably, noncontrast magnetic resonance lymphangiography and dynamic contrast-enhanced magnetic resonance lymphangiography, can now depict lymphatic anatomy and function in all major lymphatic compartments and are essential for modern therapy planning. Based on the new pathophysiologic understanding of lymphatic flow disorders, innovative minimally invasive procedures have been invented during the last few years with promising results. Abnormal lymphatic flow can now be redirected with catheter-based interventions like thoracic duct embolization, selective lymphatic duct embolization, and liver lymphatic embolization. Lymphatic drainage can be improved through surgical or interventional techniques such as thoracic duct decompression or lympho-venous anastomosis.

## Highlights

• The lymphatic system plays a central role in protein-losing enteropathy, plastic bronchitis, postoperative chylothorax, and chylous ascites.
• Noncontrast magnetic resonance lymphangiography and dynamic contrast-enhanced magnetic resonance lymphangiography can depict lymphatic anatomy and function.
• Abnormal lymphatic flow can now be redirected with catheter-based interventions like thoracic duct embolization, selective lymphatic duct embolization, and liver lymphatic embolization.
• Lymphatic drainage can be improved through surgical or interventional techniques such as thoracic duct decompression or lympho-venous anastomosis.

## Central Illustration

**Figure:**
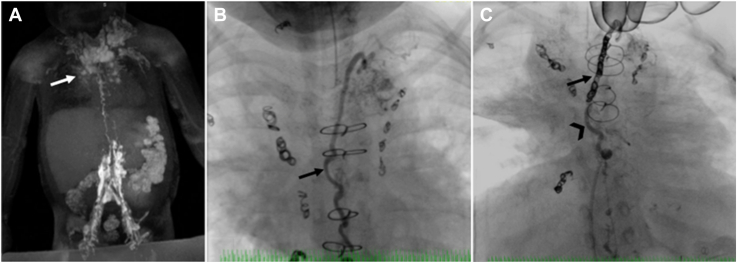

## Introduction

Congenital heart disease is associated with a variety of lymphatic flow disorders like chylothorax, plastic bronchitis, and protein-losing enteropathy.^1^, ^2^, ^3^ Hemodynamic alterations especially in single ventricle physiology, but also in congestive heart failure, make these individuals prone to lymphatic congestion and lymphatic insufficiency.^4^ In some patients, the overburdened lymphatic system finally decompresses into extra lymphatic compartments leading to plastic bronchitis, protein-losing enteropathy, or other lymphatic flow disorders that are associated with high morbidity and mortality.^5^ Treatment options were historically limited.

Thanks to the development of novel imaging modalities during the last decades, pathophysiologic mechanisms behind these diseases have been revealed.^6^ Abnormal lymphatic channels and retrograde lymphatic flow can now be detected in all major lymphatic compartments. Based on these new insights, new minimally invasive procedures have now been developed with promising results. This article aims to summarize current concepts of lymphatic flow disorders in patients with congenital heart disease, new lymphatic imaging modalities, and interventional techniques.^7^

## The lymphatic system: Anatomy and physiology

The lymphatic system is an integral part of the human cardiovascular system. It is universally distributed in the body and drains excess interstitial fluid, which is constantly generated in peripheral organs and tissue, into collecting vessels that pump it back to the blood.^8^^,^^9^ It thereby fulfills a central role in fluid balance and tissue homeostasis with far-reaching consequences on the microenvironment and cell function. In addition, it aids in transportation of micronutrients and intestinal absorption of long-chain fatty acids and is essential for immune regulation.^10^

Lymphatic anatomy is complex and variable.^11^, ^12^, ^13^ The lymphatic capillaries (initial lymphatics) are made of a network of thin-walled, blind-ended channels that collect the excess interstitial fluid. Lymph production is regulated by hydrostatic and oncotic pressures that are described by the modified Frank-Starling equation. Initial lymphatics further drain into precollecting vessels that merge to form collecting lymphatic vessels. Larger secondary collecting vessels finally enter the thoracic duct (TD) or the right lymphatic duct, via multiple tributaries.^14^

The TD is the largest lymphatic vessel in the human body. It usually commences with a fusiform dilation, the cisterna chyli, which is situated paravertebrally and to the right of the aorta at the level of T11-L1. The cisterna chyli collects lymph from the lower extremities, the liver, and the mesentery via both lumbar trunks and the intestinal lymphatic duct. The TD then ascends just anterior to the vertebral bodies into the posterior mediastinum, where it receives lymphatic flow from intrathoracic organs including both lungs and the heart. Finally, the TD runs superiorly to the neck where it enters the large veins usually on the left side.^15^ The lympho-venous junction is mostly in the proximity of the venous angle of the internal jugular vein and the subclavian vein. A bicuspid semilunar valve or ostial type valve usually sits at the mouth of the TD outlet but can be absent. It prevents backward filling of blood into the lymphatic vessels and regulates lymphatic flow. Multiple anatomical and imaging studies have demonstrated anatomic variability of both the course and the termination of the TD.^16^, ^17^, ^18^, ^19^ Apart from the thoracic duct and the right lymphatic duct, there can be multiple lympho-venous connections at different sites in the abdomen or thorax. In addition, lympho-pulmonary vein connections can be present in pathophysiologic conditions like in plastic bronchitis patients.

Lymphatic flow is usually centripetally. It is the result of a combination of intrinsic and extrinsic factors. Coordinated contractions of specialized muscle cells that surround the larger lymphatic channels are most important for lymph transportation. The negative intrathoracic pressure exerted during inspiration as well as contractions of surrounding muscles may aid in lymph flow generation. Valves that are interspersed in larger lymphatic vessels, assure unidirectional flow.^20^^,^^21^ The section between 2 valves is termed lymphangion. This functional unit serves as a primitive heart that propels the lymph further.^22^^,^^23^ Thoracic duct flow is approximately 2 to 3 liters a day under normal conditions but can increase up to 30-fold in patients with lymphatic flow disorders. The liver and intestine are the single most important contributors and generate about 80% of TD lymph together.^7^

## Lymphatic insufficiency in congenital heart disease

The lymphatic system plays a central role in some of the most devastating complications associated with congenital heart defects. Diseases like protein-losing enteropathy, plastic bronchitis, postoperative chylothorax, and chylous ascites are now proven to be lymphatic in origin.^1^^,^^10^ The etiology of these disorders is likely multifactorial. Alterations in hemodynamics as well as a congenital or genetic susceptibility with possibly anatomic variations of the lymphatic system make certain individuals prone to lymphatic dysfunction and finally lead to the development of lymphatic flow disorders.^7^

One of the most severe congenital heart defects is the spectrum of hypoplastic left heart syndrome and other functional univentricular heart defects that cannot be corrected with a biventricular repair. The Fontan operation that was introduced 50 years ago is currently the only surgical strategy that allows these individuals to survive. Hemodynamic alterations after the operation are inevitable and consist of an unphysiologically elevated central venous pressure, loss of pulsatile pulmonary flow, and a chronic reduced cardiac output with far-reaching consequences on many organs including the lymphatic system.^24^ The chronically elevated central venous pressure is maybe the single most important factor for lymphatic dysfunction. It leads to an increased efflux of fluid from the vasculature into the interstitial space and thereby stimulates lymph production, especially in the liver and the mesenteries. At the same time, lymphatic afterload is increased and alters lymph drainage capacity at the lympho-venous junction. In addition, lymphatic congestion can lead to dilation of lymphatic vessels and thereby affects valve function and lymph propulsion.^4^^,^^25^ This has been demonstrated recently in a study using near-infrared fluoroscopic imaging. It showed diminished lymphatic pumping, decreased lymphatic pumping pressure, and a higher contraction frequency in the lower extremities of Fontan patients.^26^ Other factors that might contribute to distinct lymphatic diseases in some patients are altered mesenteric vascular resistance and inflammation.^1^

## Lymphatic flow disorders in congenital heart disease

Lymphatic flow disorders frequently manifest in the thorax, the abdomen, or as multicompartment lymphatic failure.^7^ In the chest, postoperative chylothorax affects between 2% and 5% of patients after cardiothoracic surgery and leads to increased length of hospital stay and increased risk of in-hospital mortality. Based on dynamic contrast magnetic resonance lymphangiography and inguinal lymphangiography, 3 distinct etiologies can be distinguished: traumatic leak, pulmonary lymphatic perfusion syndrome, and central lymphatic flow disorder.^3^^,^^27^

Plastic bronchitis is another manifestation of lymphatic insufficiency in the thorax. Approximately 4% to 14% of Fontan patients will suffer from this complication. It is characterized by the formation of rubbery-like branching casts that can lead to airway obstruction and life-threatening asphyxia. Patients frequently present with chronic cough, wheezing, and dyspnea. The diagnosis can be confirmed with bronchoscopy or when casts are expectorated.^28^^,^^29^ In most patients, retrograde perfusion of the peribronchial lymphatic networks via abnormal lymphatic channels that arise from the TD can be detected. In some cases, hepatopulmonary connections may be the cause.^7^

Protein-losing enteropathy is the most common lymphatic flow disorder in the abdomen. It affects 5% to 12% of Fontan patients and is diagnosed when α1-antitrypsin is elevated in the stool and patients show a variable degree of hypoalbuminemia, hypogammaglobulinemia, and lymphopenia. Patients typically present with peripheral edema, ascites, pleural or pericardial effusions, and abdominal complaints like diarrhea, abdominal bloating, and pain.^30^^,^^31^ The etiology of protein-losing enteropathy in all single ventricle patients is abnormal perfusion of the duodenum via hepatoduodenal channels and in some cases via increased mesenteric production.^7^

Chylous and nonchylous ascites are less frequently seen in children but may affect up to 30% of adult Fontan patients. Truly chylous ascites is rare. It can result from traumatic lymphatic leakage. In nonchylous ascites, the etiology is often multifactorial and includes mesenteric lymphatic congestion, portal hypertension, hypoalbuminemia, renal dysfunction, and congestive heart failure with low cardiac output.

Multicompartment lymphatic failure is the most severe manifestation of lymphatic insufficiency and is diagnosed when at least 2 compartments including the thorax, abdomen, and soft tissue are involved.^7^

## Modern lymphatic imaging techniques

The development of novel lymphatic imaging techniques was paramount for the current understanding of lymphatic flow disorders and the development of innovative interventional techniques. Historically, pedal lymphangiography and lymphoscintigraphy were standard but both modalities were difficult to perform and lacked spatial or temporal resolution; therefore, they are rarely used nowadays. The introduction of noncontrast magnetic resonance lymphangiography and dynamic contrast magnetic resonance lymphangiography during the last decades has overcome these limitations and allowed a thorough assessment of lymphatic anatomy and function in the modern area.^6^

Noncontrast, heavily T2-weighted magnetic resonance lymphangiography is easy to perform and can be implemented to depict lymphatic anatomy and screen for lymphatic abnormalities in the neck and the thorax. It is usually the first step in preinterventional workup and consists of sequences that depict areas of slow-moving nonbloody fluids resembling lymphatic tissue. Based on this imaging modality, 4 types of lymphatic abnormalities can be distinguished in patients before the Fontan operation that have been shown to correlate with postoperative risk.^32^

For the assessment of lymphatic function and flow, dynamic MRI sequences that are gathered after the application of contrast media into lymphatic channels are usually the next step. This modality has been termed dynamic contrast (contrast-enhanced) magnetic resonance lymphangiography.

**Figure 1:**
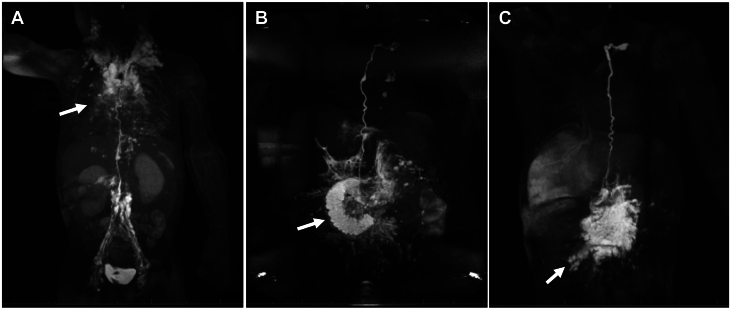
(**A**) Maximal intensity projection coronal projection of intranodal dynamic contrast magnetic resonance lymphangiography in a patient with plastic bronchitis showing bilateral pulmonary lymphatic perfusion syndrome (arrow). (**B**) Maximal intensity projection coronal projection of IH-DCMRL in a patient with protein-losing enteropathy demonstrating leak into the duodenum (arrow). (**C**) IM-DCMRL in a patient with retrograde mesenteric flow (arrow).

Dynamic contrast (contrast-enhanced) magnetic resonance lymphangiography can only visualize lymphatic structures that are in the pathway of the contrast medium. To investigate all major lymphatic compartments in the trunk, 3 different types of dynamic contrast magnetic resonance lymphangiography are now available: intranodal dynamic contrast magnetic resonance lymphangiography, intrahepatic dynamic contrast magnetic resonance lymphangiography, and intramesenteric dynamic contrast magnetic resonance lymphangiography (Figure 1).

Intranodal dynamic contrast magnetic resonance lymphangiography is the most widely used modality and it is essential to investigate the central lymphatic flow from the lower extremities through the TD into the venous system as well as abnormal lymphatic channels that branch off these structures.^33^ This is especially important in patients with plastic bronchitis chylopericardium and chylothorax, where abnormal retrograde lymphatic flow is most often originating from the TD or its tributaries.^34^^,^^35^ Intranodal dynamic contrast magnetic resonance lymphangiography involves the cannulation of superficial inguinal lymph nodes on both sides with a 25-gauge spinal needle. This is usually done under ultrasound guidance. The correct position can either be verified under fluoroscopy or by ultrasound. After the needles are secured (for example, with Tegaderm [3M]) the patient is moved to the magnetic resonance imaging suite. For all dynamic contrast (contrast-enhanced) magnetic resonance lymphangiography techniques, an akinetic transfer to the magnetic resonance imaging table is essential to avoid needle dislocation. After gathering native sequences, 0.2 mL per kg of body weight of Gadolinium, which can be diluted with 1:1 or 1:2 volumes of saline is administered into both lymph nodes at a slow rate (approximately 1 mL/min). Dynamic sequences are usually started during contrast medium application and timing depends on lymph flow propagation.

The liver lymphatics can be investigated with intrahepatic dynamic contrast magnetic resonance lymphangiography. In this approach, a 25-gauge spinal needle is placed under ultrasound guidance in proximity to a branch of the portal vein. Afterward, iodine contrast is applied through the needles under fluoroscopy and the position is adjusted until the liver lymphatics are clearly seen and the needles can be secured (for example, with Tegaderm). With intrahepatic dynamic contrast magnetic resonance lymphangiography, abnormal liver lymphatic flow to the duodenum, lungs, peritoneum or mesenteries can be seen. Hepatoduodenal connections are thereby frequently depicted in protein-losing enteropathy patients. Hepatopulmonary connections are rare but characteristic of chylothorax and plastic bronchitis and hepatoperitoneal connections may be seen in ascites.^36^^,^^37^

Intramesenteric dynamic contrast magnetic resonance lymphangiography is most challenging and involves the cannulation of mesenteric lymphatic ducts or lymph nodes in the anterior portion of the small intestine with a 25-gauge spinal needle. This is done similar to the intrahepatic approach using ultrasound and fluoroscopy. Intramesenteric lymphatic access is important for detecting peritoneal leaks and it can show duodenal leaks not seen in the intrahepatic approach.^38^

Ultrasound is another modality that can aid in the assessment of lymphatic structures and function. With gray-scale ultrasound, the terminal part of the TD including the outlet can be visualized.^39^ Microbubble ultrasound contrast agents can be applied to the lymphatic system whether via intranodal or intrahepatic needles before or after dynamic contrast magnetic resonance lymphangiography. This allows direct visualization of lymphatic flow into the venous system and has been used successfully to confirm TD patency in a small study.^40^

Conventional fluoroscopic lymphangiography techniques are less frequently used but still have a role during lymphatic interventions or when there are contraindications for magnetic resonance lymphangiography. Lymphatic access is identical to the dynamic contrast magnetic resonance lymphangiography techniques, but instead of Gadolinium, an iodinated water-soluble contrast agent or lipiodol is administered to visualize the lymphatic structures. In general, opacification of the central lymphatic system is usually profoundly poorer during fluoroscopic lymphangiography and lipiodol usage has been associated with some severe complications including cerebral stroke.^41^^,^^42^ In all patients, a potential right to left shunt should therefore be ruled out or closed before lipiodol administration.^43^

## Innovative lymphatic interventional procedures in the management of lymphatic disease

Modern management of lymphatic insufficiency is multidisciplinary. It involves medical, interventional, and surgical strategies.^7^ The first step is a thorough assessment of hemodynamics and lymphatic anatomy and function as described before. A cardiac catheterization should therefore be performed in all individuals with suspected lymphatic abnormalities to search for systemic obstruction and to assess atrioventricular valve competence, pulmonary anatomy, and pulmonary vascular resistance. If possible, all anatomic alterations have to be addressed as the elimination of stenosis can improve lymphatic insufficiency markedly. A Holter electrocardiogram can be useful to detect relevant conduction abnormalities, and a gastroscopy and duodenoscopy can be performed to exclude differential diagnosis in patients with enteric protein loss.^44^

Basic medical management includes high-dose spironolactone, other diuretics, and pulmonary vasodilators and should be optimized. These medications are aimed at reducing venous and lymphatic congestion to decrease pulmonary resistance and treat edemas and effusions. In protein-losing enteropathy, serial albumin replacements may become necessary in some patients to increase oncotic pressure and alleviate edemas. In addition, oral controlled-release budesonide may be beneficial to reduce protein loss. In plastic bronchitis, bronchodilators, chest physiotherapy, inhaled mucolytics, and inhaled corticosteroids have been tried in some patients with success. Nebulized tissue plasminogen activator can be used to clear obstructive airway casts in acutely symptomatic individuals and bronchoalveolar lavage can be lifesaving in acute airway obstruction.^1^

During the last decades, a series of new minimally invasive and surgical techniques have been invented to target abnormal lymphatic channels and improve lymphatic drainage. They can be divided into 2 main groups, those that are meant to decompress the entire lymphatic system such as lympho-venous anastomosis and surgical or interventional TD decompression, and those that are aimed to seal abnormal lymphatic vessels like TD embolization, selective lymphatic duct embolization, and liver lymphatic embolization.^36^^,^^45^, ^46^, ^47^

Before considering any intervention, potential right to left shunts should be considered in patients at risk because this imposes the risk of embolization of ethiodized oil or glue into the systemic circulation during the procedures, leading to stroke. When shunts are present, they can be closed temporarily or permanently to reduce the risk. Veno-venous collateral vessels near the lympho-venous junction or lympho-pulmonary vein connections can be embolized for example before an open fenestration can be occluded in Fontan patients with a balloon during the intervention. Especially lympho-pulmonary vein connections are difficult to rule out; so, the amount of lipiodol should be kept as low as possible. Balloon occlusion on the other hand might itself increase the risk of thrombus formation because during an intervention anticoagulation is prohibited.^43^

**Figure 2:**
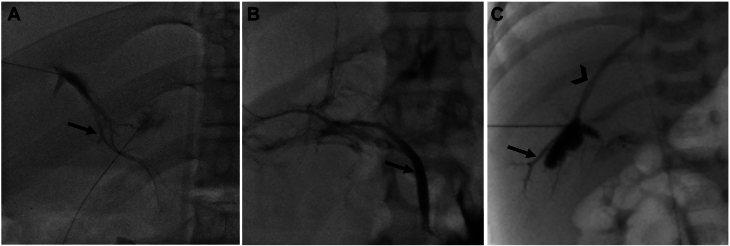
**Fluoroscopy images in theanterior-posteriorprojection of IH lymphangiography**. (**A**) Liver lymphatic channels (arrow), (**B**) biliary tree (arrow), (**C**) portal veins (arrow), and hepatic veins (arrowhead).

Another complication that has to be considered during intervention is the unintentional gluing of nontargeted structures. This can potentially be veins, the bile duct, the pancreatic duct, or the TD itself in selective lymphatic duct embolization (Figure 2). To minimize the risk, in addition to the knowledge of different image patterns seen on conventional fluoroscopy, blue dye can be applied and abnormal connections to the lung and the duodenum can be confirmed by endoscopy.

After the intervention, gastrointestinal bleeding from repeated transabdominal punctures, pancreatitis, and a systemic inflammatory response can occur.

### Minimally invasive interventions

Ethiodized oil lymphatic embolization is a relatively easy but unselective minimally invasive interventional approach. The technique is similar to intranodal dynamic contrast magnetic resonance lymphangiography. After inguinal lymph node access is gained under ultrasound guidance, lipiodol, an iodinated contrast agent, is administered at a slow rate. It is usually used to visualize the central lymphatic system during conventional intranodal lymphangiography but has also been shown to have therapeutic effects. Lipiodol is an oily substance that has a high viscosity and can lead to sclerosis of small fistulas over a period of weeks. It has recently been shown to be effective in the treatment of neonatal chylothorax.^48^^,^^49^

Thoracic duct embolization was first described by Cope in 1998 and is now used in the treatment of chylothorax, chylopericardium, and plastic bronchitis.^10^^,^^50^ It was developed as a minimally invasive alternative to surgical TD ligation. Central lymphatic access can be gained from either an anterograde percutaneous transabdominal approach or from a retrograde approach.^43^ The antegrade approach involves percutaneous transabdominal puncture of either the cisterna chyli or another larger retroperitoneal lymphatic vessel that is opacified using intranodal lymphangiography. Usually, a 22- to 25-gauge spinal needle is used to insert a 0.010" to 0.018" guide wire into the TD that is then further advanced cranially up to the lympho-venous junction. Once a stable wire position is gained, the needle is removed and a small microcatheter is advanced over the wire into the TD. After removal of the wire, imaging of the TD and subsequent embolization can be done with either a cyanoacrylate-based glue alone or in combination with endovascular micro coils (Central Illustration). To prevent the efflux of glue into the venous system, the TD outlet can be compressed externally or occluded with a transvenous balloon. In patients with elevated central venous pressure like in a Fontan circulation, complete occlusion of the TD should be avoided because it can lead to the development of downstream lymphatic complications like protein-losing enteropathy or ascites.

**Central Illustration:**
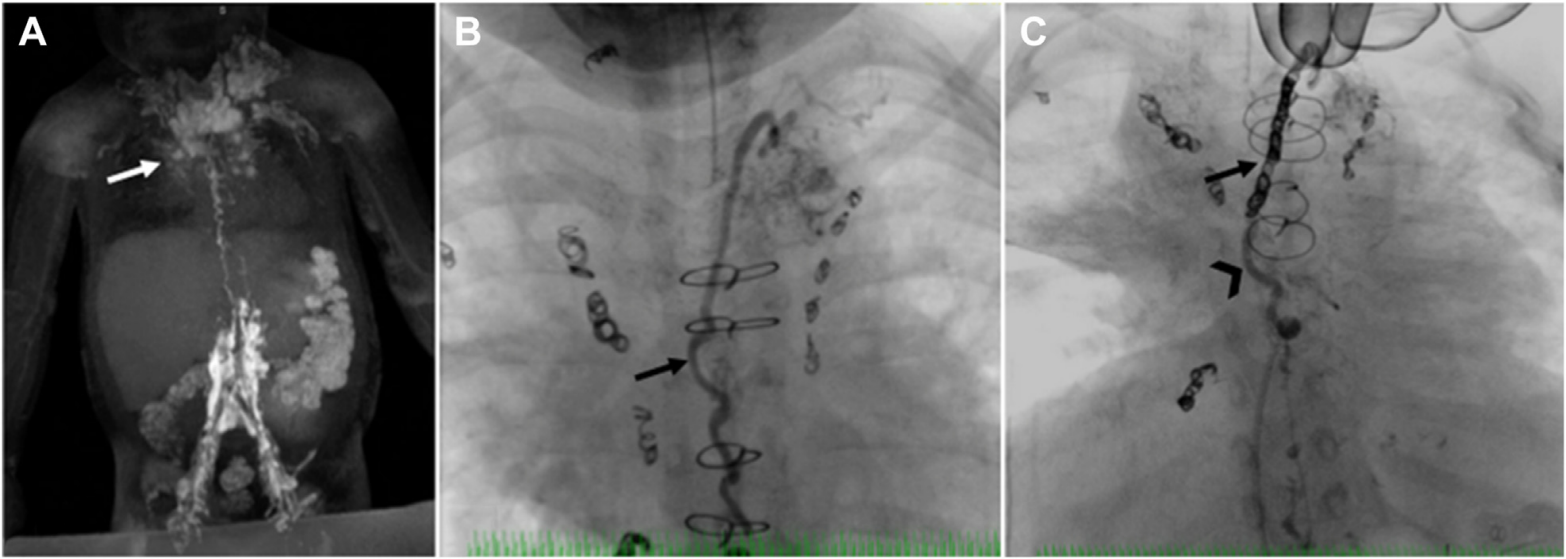
Novel lymphatic imaging techniques (ie, dynamic contrast magnetic resonance lymphangiography) and minimally invasive procedures (thoracic duct embolization) used for successful treatment of lymphatic complications (plastic bronchitis and chylothorax) in a patient with a congenital heart defect. (**A**) Maximal intensity projection coronal projection of intranodal-DCMRL showing bilateral pulmonary lymphatic perfusion syndrome (arrow). (**B**) Fluoroscopic lymphangiography in the anterior-posterior projection shows a thoracic duct (arrow) and bilateral pulmonary lymphatic perfusion syndrome. (**C**) Coil (arrow) and glue (arrowhead) seen in the thoracic duct after thoracic duct embolization.

**Figure 3:**
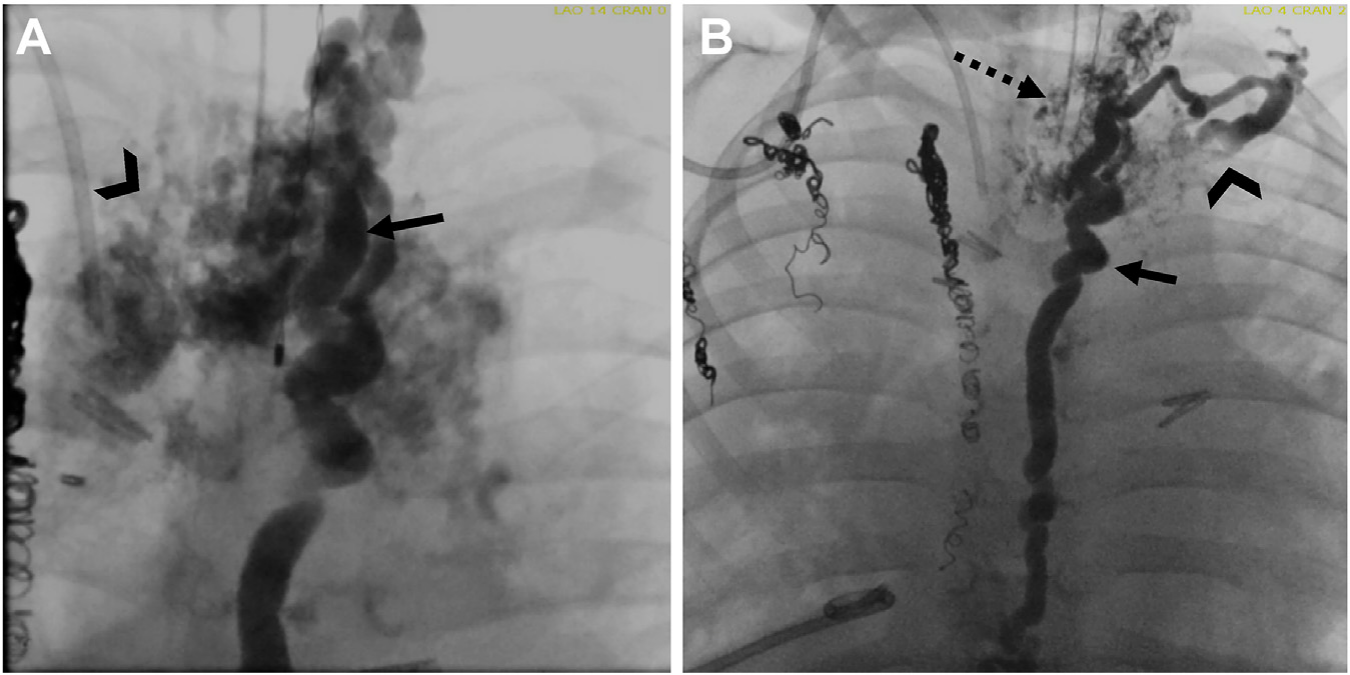
(**A**) Fluoroscopic lymphangiography in the anterior-posterior projection shows the dilated thoracic duct (arrow) and mediastinal as well as bilateral pulmonary lymphatic perfusion syndrome (arrowhead). (**B**) The thoracic duct (arrow) is seen after selective lymphatic duct embolization of the abnormal channels (hashed arrow) showing no residual perfusion of the mediastinum or lungs with the duct draining into the vein (arrowhead).

Selective lymphatic duct embolization is a more recently developed technique and is now preferred whenever possible.^7^ It allows embolization of lymphatic collaterals that are branching from the TD and responsible for retrograde lymphatic flow which is frequently seen in plastic bronchitis, chylothorax, and chylopericardium.^35^ This technique is an advancement of TD embolization and is performed similarly. After gaining thoracic duct access as described before, the microcatheter is further advanced from the TD into the targeted channel that is then embolized with coils and/or glue (Figure 3). To prevent the reflux of glue into the TD, a second microcatheter can be placed in the TD and flushed with glucose during glue administration. Proper priming of the system with 5% dextrose is essential and an appropriate dilution of the glue with ethiodized oil determines the length of the segment that is finally sealed (Supplemental Videos 1 and 2). Normally 1:1 dilution for short segment embolization and 1:3 dilution for long segment embolization is feasible. In some cases, the abnormal lymphatic vessel can be punctured directly and then can be embolized through the needle. If multiple or small abnormal channels branch from the TD, covered stents can be placed in the TD to exclude abnormal lymphatic flow.^35^

**Figure 4:**
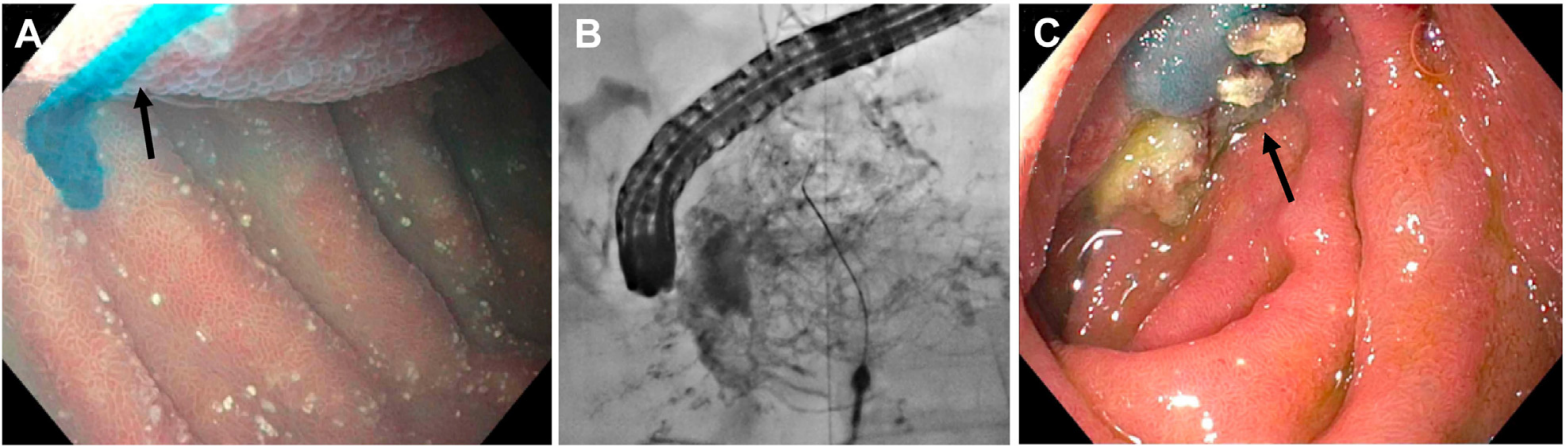
(**A**) Blue dye injected into the liver lymphatic channels is seen via endoscopy leaking into the duodenal lumen (arrow). (**B**) Fluoroscopy in the anterior-posterior projection after glue embolization of periduodenal lymphatic networks. (**C**) Glue is seen inside the duodenal lumen after embolization (arrow).

The liver lymphatics play a major role in protein-losing enteropathy and ascites but can also be affected in chylothorax and plastic bronchitis.^37^^,^^38^ The technique of liver lymphatic embolization is similar to intrahepatic dynamic contrast magnetic resonance lymphangiography using a bimodal approach (Figure 4). At first, a 25-gauge spinal needle is placed under ultrasound guidance in proximity to a branch of the portal vein, and the position is adjusted under fluoroscopy until abnormal lymphatic collaterals are visualized. To confirm correct needle position in a feeding vessel, isosulfan blue can be injected through the needle. In protein-losing enteropathy patients with hepatoduodenal fistulas, an endoscope is placed in the periampullary region at the same time to demonstrate leakage of blue dye into the duodenum and to rule out contrast exiting from the ampulla. In patients with plastic bronchitis, a hepatopulmonary fistula can be confirmed similarly with bronchoscopy.

### Thoracic duct decompression

**Figure 5:**
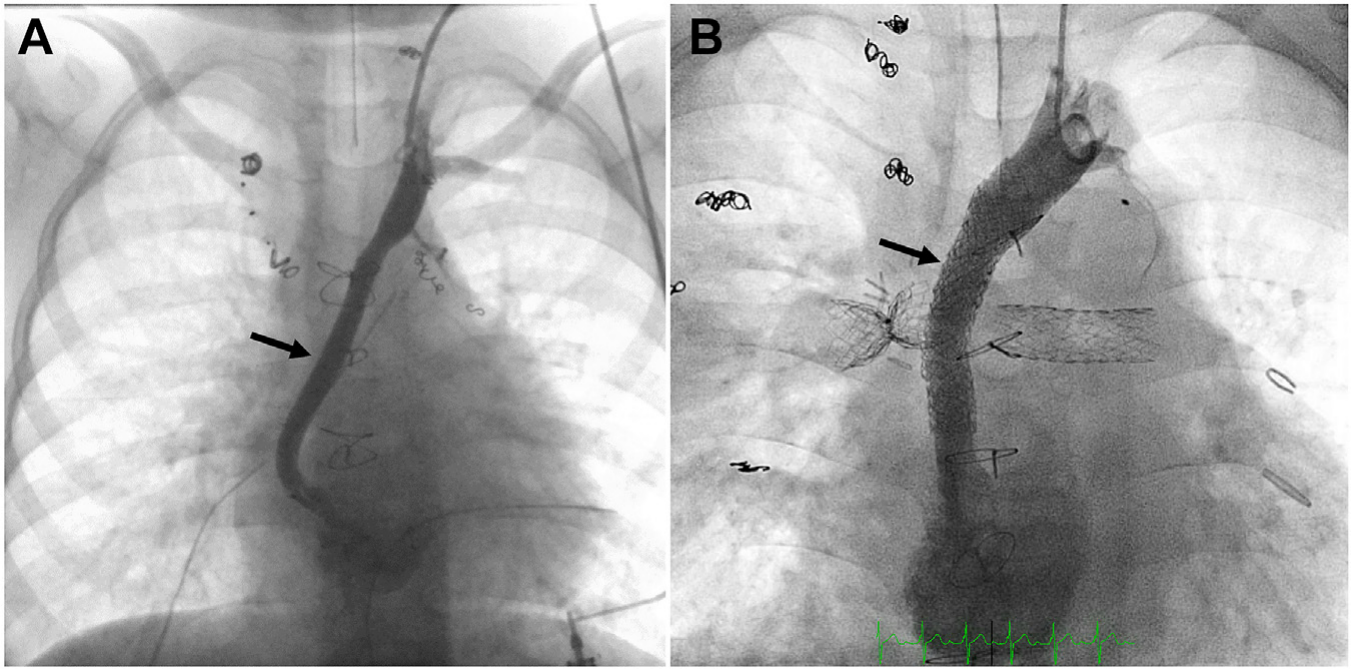
(**A**) Contrast injection in the left internal jugular vein in the anterior-posterior projection after surgical thoracic duct decompression showing a patent connection of the innominate vein to the atrium (arrow), (**B**) contrast injection in the left internal jugular vein after percutaneous TD decompression showing a patent connection of the stent to the atrium (arrow).

There are now several approaches that are meant to improve lymph drainage. In 2013, Victor Hraska first described a surgical procedure to decompress the TD.^51^ He used the innominate vein that was divided upstream to the junction with the right internal jugular vein and then reanastomosed it to the common atrium to redirect lymphatic flow into the lower pressure environment of the atrium. Several case series and case reports have been published since then using his operation strategy with slight modifications. In some patients, an interposition graft was necessary if there was no adequate length of the innominate vein for primary turn-down (Figure 5).^45^

A similar percutaneous transcatheter approach was first reported in 2 patients where a connection between a left superior vena cava and a left atrium was created with covered stents.^52^ The technique has then been modified to create an extravascular shunt and allow TD decompression in many different anatomies.^53^

An unobstructed TD outflow and a low atrial pressure level are essential for therapeutic success and have to be confirmed before the decompression procedure. Furthermore, prior to decompression, embolization of larger lymphatic leaks might be needed. The iatrogenic left to right shunt may decrease oxygen saturation after the intervention but is generally well tolerated. If profound desaturation occurs, hypoxemia can be improved with banding of the internal jugular vein.^54^

In patients where the TD outlet is obstructed, microsurgical techniques have been used to create lympho-venous anastomosis. A successful thoracic duct-to-vein anastomosis was recently described in 2 patients with therapy refractory chylothoraxes.^55^ The TD was visualized with a transcatheter approach, divided distal to the leakage site, and then anastomosed end-to-end or end-to-side to an adjacent vein or venule. If possible, a larger vein, such as the external jugular vein or a branch of this, can be used instead.

## Conclusion

Patients with congenital heart defects especially with single ventricle physiology are prone to lymphatic dysfunction and the development of lymphatic complications like protein-losing enteropathy, plastic bronchitis, chylothorax, ascites, or multicompartment lymphatic failure.

Noncontrast and dynamic contrast magnetic resonance lymphangiography have now become the imaging modalities of choice in the diagnosis of lymphatic insufficiency in these patients and allow a thorough assessment of anatomy and function of the main lymphatic compartments through intranodal, intrahepatic, and intramesenteric lymphatic imaging. Based on individual pathophysiology, minimally invasive procedures can now be provided to redirect pathologic central lymphatic flow, occlude pathologic lymphatic channels, and improve lymphatic drainage through decompression procedures. The results of all these new techniques are overall promising, but further evidence is needed for proper patient selection and long-term outcomes.

## Supplementary material

Supplemental Video 1 **Lymphatic imaging in a** **p****atient with****chylothorax****before intervention**. Lymphatic imaging in a 3-year-old patient with multicompartment lymphatic failure and therapy-resistant bilateral chylothorax before lymphatic intervention. There is a big tortuous fistula arising from the thoracic duct to the left lung. Note the 2 microcatheters placed in the proximal and distal thoracic ducts.

Supplemental Video 2 **Lymphangiogram in a** **p****atient with****chylothorax****after intervention**. Lymphangiogram in a 3-year-old patient with multicompartment lymphatic failure and therapy-resistant bilateral chylothorax after glue administration. The fistula is filled with a glue-lipiodol mixture. Note that there is still 1 microcatheter placed in the distal thoracic duct to confirm thoracic duct patency and prove the efficacy of fistula closure.

## References

[1] Rychik J., Atz A.M., Celermajer D.S. (2019). Evaluation and management of the child and adult with Fontan circulation: A scientific statement from the American Heart Association. Circulation.

[2] Villena V., de Pablo A., Martín-Escribano P. (1995). Chylothorax and chylous ascites due to heart failure. Eur Respir J.

[3] Mery C.M., Moffett B.S., Khan M.S. (2014). Incidence and treatment of chylothorax after cardiac surgery in children: analysis of a large multi-institution database. J Thorac Cardiovasc Surg.

[4] Menon S., Chennapragada M., Ugaki S., Sholler G.F., Ayer J., Winlaw D.S. (2017). The lymphatic circulation in adaptations to the Fontan circulation. Pediatr Cardiol.

[5] Ghosh R.M., Griffis H.M., Glatz A.C. (2020). Prevalence and cause of early Fontan complications: does the lymphatic circulation play a role?. J Am Heart Assoc.

[6] Dori Y. (2016). Novel lymphatic imaging techniques. Tech Vasc Interv Radiol.

[7] Dori Y., Smith C.L. (2022). Lymphatic disorders in patients with single ventricle heart disease. Front Pediatr.

[8] Skandalakis J.E., Skandalakis L.J., Skandalakis P.N. (2007). Anatomy of the lymphatics. Surg Oncol Clin N Am.

[9] Hsu M.C., Itkin M. (2016). Lymphatic anatomy. Tech Vasc Interv Radiol.

[10] Tomasulo C.E., Chen J.M., Smith C.L., Maeda K., Rome J.J., Dori Y. (2022). Lymphatic disorders and management in patients with congenital heart disease. Ann Thorac Surg.

[11] Kausel H.W., Reeve T.S., Stein A.A., Alley R.D., Stranahan A. (1957). Anatomic and pathologic studies of the thoracic duct. J Thorac Surg.

[12] Cha E.M., Sirijintakarn P. (1976). Anatomic variation of the thoracic duct and visualization of mediastinal lymph nodes. A lymphographic study. Radiology.

[13] Tammela T., Alitalo K. (2010). Lymphangiogenesis: molecular mechanisms and future promise. Cell.

[14] Null M., Arbor T.C., Agarwal M. (2023). StatPearls.

[15] Hematti H., Mehran R.J. (2011). Anatomy of the thoracic duct. Thorac Surg Clin.

[16] Gottlieb M.I., Greenfield J. (1956). Variations in the terminal portion of the human thoracic duct. AMA Arch Surg.

[17] Johnson O.W., Chick J.F.B., Chauhan N.R. (2016). The thoracic duct: clinical importance, anatomic variation, imaging, and embolization. Eur Radiol.

[18] Brotons M.L., Bolca C., Fréchette E., Deslauriers J. (2012). Anatomy and physiology of the thoracic lymphatic system. Thorac Surg Clin.

[19] O’Hagan L.A., Windsor J.A., Itkin M., Russell P.S., Phillips A.R.J., Mirjalili S.A. (2021). The lymphovenous junction of the thoracic duct: A systematic review of its structural and functional anatomy. Lymph Res Biol.

[20] Scallan J.P., Zawieja S.D., Castorena-Gonzalez J.A., Davis M.J. (2016). Lymphatic pumping: mechanics, mechanisms and malfunction. J Physiol.

[21] Li H., Mei Y., Maimon N., Padera T.P., Baish J.W., Munn L.L. (2019). The effects of valve leaflet mechanics on lymphatic pumping assessed using numerical simulations. Sci Rep.

[22] Venugopal A.M., Stewart R.H., Laine G.A., Dongaonkar R.M., Quick C.M. (2007). Lymphangion coordination minimally affects mean flow in lymphatic vessels. Am J Physiol Heart Circ Physiol.

[23] van Helden D.F. (2014). The lymphangion: A not so “primitive” heart. J Physiol.

[24] Rychik J. (2016). The relentless effects of the Fontan paradox. Semin Thorac Cardiovasc Surg Pediatr Card Surg Annu.

[25] RochéRodríguez M., DiNardo J.A. (2022). The lymphatic system in the Fontan patient—pathophysiology, imaging, and interventions: what the anesthesiologist should know. J Cardiothorac Vasc Anesth.

[26] Mohanakumar S., Telinius N., Kelly B. (2019). Morphology and function of the lymphatic vasculature in patients with a Fontan circulation. Circ Cardiovasc Imaging.

[27] Savla J.J., Itkin M., Rossano J.W., Dori Y. (2017). Post-operative chylothorax in patients with congenital heart disease. J Am Coll Cardiol. Elsevier United States.

[28] Caruthers R.L., Kempa M., Loo A. (2013). Demographic characteristics and estimated prevalence of Fontan-associated plastic bronchitis. Pediatr Cardiol.

[29] Bearl D.W., Cantor R., Koehl D. (2021). Fontan-associated plastic bronchitis waitlist and heart transplant outcomes: a PHTS analysis. Pediatr Transplant.

[30] Rychik J. (2007). Protein-Losing enteropathy after Fontan operation. Congenit Heart Dis.

[31] Itkin M., Piccoli D.A., Nadolski G. (2017). Protein-losing enteropathy in patients with congenital heart disease. J Am Coll Cardiol.

[32] Biko D.M., DeWitt A.G., Pinto E.M. (2019). MRI evaluation of lymphatic abnormalities in the neck and thorax after Fontan surgery: relationship with outcome. Radiology.

[33] Ramirez-Suarez K.I., Tierradentro-Garcia L.O., Smith C.L. (2022). Dynamic contrast-enhanced magnetic resonance lymphangiography. Pediatr Radiol.

[34] Itkin M., Chidekel A., Ryan K.A., Rabinowitz D. (2020). Abnormal pulmonary lymphatic flow in patients with paediatric pulmonary lymphatic disorders: diagnosis and treatment. Paediatr Respir Rev.

[35] Dori Y., Keller M.S., Rome J.J. (2016). Percutaneous lymphatic embolization of abnormal pulmonary lymphatic flow as treatment of plastic bronchitis in patients with congenital heart disease. Circulation.

[36] Lemley B.A., Biko D.M., Dewitt A.G. (2021). Intrahepatic dynamic contrast-enhanced magnetic resonance lymphangiography: potential imaging signature for protein-losing enteropathy in congenital heart disease. J Am Heart Assoc.

[37] Biko D.M., Smith C.L., Otero H.J. (2019). Intrahepatic dynamic contrast MR lymphangiography: initial experience with a new technique for the assessment of liver lymphatics. Eur Radiol.

[38] Dori Y., Smith C.L., DeWitt A.G. (2020). Intramesenteric dynamic contrast pediatric MR lymphangiography: initial experience and comparison with intranodal and intrahepatic MR lymphangiography. Eur Radiol.

[39] Seeger M., Bewig B., Günther R. (2009). Terminal part of thoracic duct: high-resolution US imaging. Radiology.

[40] Mejia E.J., Otero H.J., Smith C.L. (2020). Use of contrast-enhanced ultrasound to determine thoracic duct patency. J Vasc Interv Radiol.

[41] Sheybani A., Gaba R.C., Minocha J. (2015). Cerebral embolization of ethiodized oil following intranodal lymphangiography. Semin Intervent Radiol.

[42] Kirschen M.P., Dori Y., Itkin M., Licht D.J., Ichord R., Vossough A. (2016). Cerebral lipiodol embolism after lymphatic embolization for plastic bronchitis. J Pediatr.

[43] Majdalany B.S., Sanogo M.L., Pabon-Ramos W.M. (2020). Complications during lymphangiography and lymphatic interventions. Semin Intervent Radiol.

[44] Bauer C., Tulzer G. (2020). Are children with protein-losing enteropathy after the Fontan operation at increased risk of cytomegalovirus enteropathy? A report of two cases. Cardiol Young.

[45] Hraska V., Hjortdal V.E., Dori Y., Kreutzer C. (2021). Innominate vein turn-down procedure: killing two birds with one stone. JTCVS Tech.

[46] Chen E., Itkin M. (2011). Thoracic duct embolization for chylous leaks. Semin Intervent Radiol.

[47] Maleux G., Storme E., Cools B. (2019). Percutaneous embolization of lymphatic fistulae as treatment for protein-losing enteropathy and plastic bronchitis in patients with failing Fontan circulation. Catheter Cardiovasc Interv.

[48] Pinto E., Dori Y., Smith C. (2021). Neonatal lymphatic flow disorders: impact of lymphatic imaging and interventions on outcomes. J Perinatol.

[49] Haga M., Kato M., Kanno M., Shimizu M., Watanabe S. (2020). Lipiodol lymphangiography in a very low birth weight premature infant. J Pediatr Surg Case Rep.

[50] Cope C. (1998). Diagnosis and treatment of postoperative chyle leakage via percutaneous transabdominal catheterization of the cisterna chyli: A preliminary study. J Vasc Interv Radiol.

[51] Hraška V. (2013). Decompression of thoracic duct: new approach for the treatment of failing Fontan. Ann Thorac Surg.

[52] Smith C.L., Hoffman T.M., Dori Y., Rome J.J. (2020). Decompression of the thoracic duct: A novel transcatheter approach. Catheter Cardiovasc Interv.

[53] Smith C.L., Dori Y., O’Byrne M.L., Glatz A.C., Gillespie M.J., Rome J.J. (2022). Transcatheter thoracic duct decompression for multicompartment lymphatic failure after Fontan palliation. Circ Cardiovasc Interv.

[54] Bauer C., Mair R., Mair R., Tulzer G. (2020). Thoracic duct decompression and jugular vein banding-an effective treatment option for protein-losing enteropathy and plastic bronchitis in severe failing Fontan circulation: a case report. Eur Heart J Case Rep.

[55] Weissler J.M., Cho E.H., Koltz P.F. (2018). Lymphovenous anastomosis for the treatment of chylothorax in infants: A novel microsurgical approach to a devastating problem. Plast Reconstr Surg.

