# Supplementary material for: Lymphatic Imaging and Intervention in Congenital Heart Disease
Source: J Soc Cardiovasc Angiogr Interv. 2023 Sep 27;3(1):101174. doi: 10.1016/j.jscai.2023.101174 (PMC11308220; doi:10.1016/j.jscai.2023.101174)
Supplement: Supplementary Data [file mmc3.docx]

Description of videos:

Video 1 : Patient with CT before intervention:

Lymphatic imaging in a 3 year old patient with multicompartment lymphatic failure and therapy resistant bilateral chylothorax before lymphatic intervention. There is a big tortuous fistula arising from the thoracic duct to the left lung. Note the two micro catheters placed in the proximal and distal thoracic duct.

Video 2 : Patient with CT after intervention:

Lymphangiogram in a 3 year old patient with multicompartment lymphatic failure and therapy resistant bilateral chylothorax after glue administration. The fistula is filled with a glue-lipiodol mixture. Note there is still one micro catheters placed in the distal thoracic duct to confirm thoracic duct patency and prove efficacy of fistula closure.
